# A systematic review of SARS-CoV-2 vaccine candidates

**DOI:** 10.1038/s41392-020-00352-y

**Published:** 2020-10-13

**Authors:** Yetian Dong, Tong Dai, Yujun Wei, Long Zhang, Min Zheng, Fangfang Zhou

**Affiliations:** 1 The State Key Laboratory for Diagnosis and Treatment of Infectious Diseases, National Clinical Research Center for Infectious diseases, Collaborative Innovation Center for Diagnosis and Treatment of Infectious Diseases, The First Affiliated Hospital, School of Medicine, Zhejiang University, Hangzhou, 310058 China; 2 Life Sciences Institute and Innovation Center for Cell Signaling Network, Hangzhou, 310058 China; 3 Institutes of Biology and Medical Science, Soochow University, Suzhou, 215123 China; 4 Anhui Anlong Gene Technology Co., Ltd, Hefei, 230041 China

**Keywords:** Vaccines, Vaccines

## Abstract

Severe acute respiratory syndrome coronavirus 2 (SARS-CoV-2) is an emerging virus that is highly pathogenic and has caused the recent worldwide pandemic officially named coronavirus disease (COVID-19). Currently, considerable efforts have been put into developing effective and safe drugs and vaccines against SARS-CoV-2. Vaccines, such as inactivated vaccines, nucleic acid-based vaccines, and vector vaccines, have already entered clinical trials. In this review, we provide an overview of the experimental and clinical data obtained from recent SARS-CoV-2 vaccines trials, and highlight certain potential safety issues that require consideration when developing vaccines. Furthermore, we summarize several strategies utilized in the development of vaccines against other infectious viruses, such as severe acute respiratory syndrome coronavirus (SARS-CoV) and Middle East respiratory syndrome coronavirus (MERS-CoV), with the aim of aiding in the design of effective therapeutic approaches against SARS-CoV-2.

## Introduction

The coronavirus disease (COVID-19) caused by severe acute respiratory syndrome coronavirus 2 (SARS-CoV-2) has posed a serious threat to public health.^1–3^ SARS-CoV-2 belongs to the Betacoronavirus of the family Coronaviridae, and commonly induces respiratory symptoms, such as fever, unproductive cough, myalgia, and fatigue.^4–6^ To better understand the virus, numerous studies have been performed, and strategies have been established with the aim to prevent further spread of COVID-19, and to develop efficient and safe drugs and vaccines.^7^ For example, the structures of viral proteins, such as the spike protein (S protein), main protease (Mpro), and RNA-dependent RNA polymerase (RdRp), have been uncovered,^8–10^ providing information for the design of drugs against SARS-CoV-2. In addition, elucidating the immune responses induced by SARS-CoV-2 is accelerating the development of therapeutic approaches. In essence, diverse small molecule drugs and vaccines are being developed to treat COVID-19. According to the World Health Organization (WHO), as of September 17, 2020, 36 vaccine candidates were under clinical evaluation to treat COVID-19, and 146 candidate vaccines were in preclinical evaluation. Given that vaccines can be applied for prophylaxis and the treatment for SARS-CoV-2 infection, in this review, we introduce the recent progress of therapeutic vaccines candidates against SARS-CoV-2. Furthermore, we summarize the safety issues that researchers may be confronted with during the development of vaccines. We also describe some effective strategies to improve the vaccine safety and efficacy that were employed in the development of vaccines against other pathogenic agents, with the hope that this review will aid in the development of therapeutic methods against COVID-19.

## Target antigen for SARS-CoV-2 vaccines

Coronaviruses (CoVs), including SARS-CoV, MERS-CoV, and SARS-CoV-2, are cytoplasmically replicating, positive-sense, single-stranded RNA viruses with four structural proteins (namely S protein, envelope (E) protein, membrane (M) protein, and nucleocapsid (N) protein).^11^ Generally, the S protein plays a crucial role in eliciting the immune response during disease progression.^12^

SARS-CoV-2 enters host cells via the same receptor, angiotensin-converting enzyme 2 (ACE2), as SARS-CoV, and the S protein is required for cell entry.^13–15^ The trimeric S protein contains two subunits, S1 and S2, which mediate receptor binding and membrane fusion, respectively. The S1 subunit contains a fragment called the receptor-binding domain (RBD) that is able to bind ACE2.^16,17^ Binding of the S protein to the ACE2 receptor triggers complex conformational changes, driving the S protein from a prefusion conformation to a postfusion conformation. The decoration of the postfusion conformation with N-linked glycans was suggested as a potential strategy for the virus to evade the host immune response.^18^ Previous studies reported that vaccines encoding SARS-CoV S protein elicited potent cellular and humoral immune responses in murine challenge models and in clinical trials.^19–21^ Similarly, the S gene is regarded as a key target for SARS-CoV-2 vaccines.^22^ The S protein of CoVs, especially the RBD, is able to induce neutralizing antibodies (NAbs) and T-cell immune responses.^23–26^ An animal study demonstrated that SARS-CoV-2 RBD-specific IgG accounted for half of the S protein-induced antibody responses.^27^ RBD-specific antibodies and T cells were also detected in the sera of discharged SARS-CoV-2-infected patients.^28^ Moreover, NAb titers were significantly correlated with the levels of anti-RBD IgG, and RBD-specific IgG titers were suggested as a surrogate of neutralization potency against SARS-CoV-2 infection.^26,28^ Furthermore, immunization with RBD was initially successful in eliciting NAbs in rodents without mediating antibody-dependent enhancemnt.^29^ Thus, RBD is a promising target for SARS-CoV-2 vaccines and previous knowledge from using RBD-based vaccines against SARS-CoV and MERS-CoV could inform the design of RBD-based SARS-CoV-2 vaccines.

**Fig. 1:**
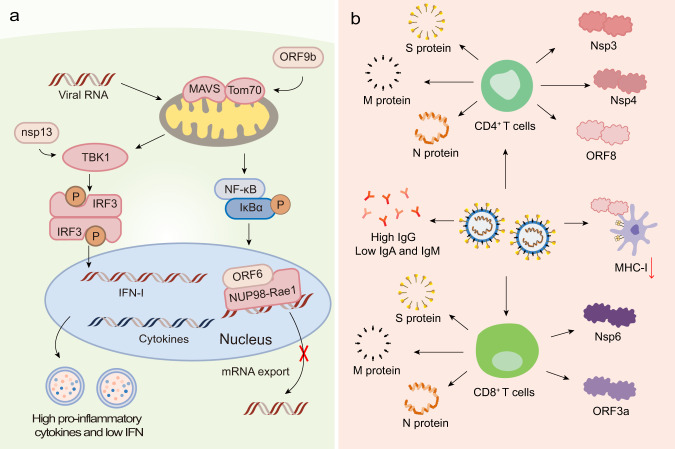
The immune responses induced by SARS-CoV-2. **a** Innate immune response. SARS-CoV-2 infection induces imbalanced host immune responses, such as low IFN-I and -III levels but high pro-inflammatory cytokines. Nsp13 of SARS-CoV-2 targets the IFN pathway by associating with TBK1. The ORF6 protein interacts with the mRNA export factor NUP98-Rae1. The ORF9b indirectly interacts with MAVS via its interaction with Tom70. **b** Adaptive immune response. CD4^+^ T-cell responses are primarily directed against the S, M, and N proteins and partially against nsp3, nsp4, and ORF8. CD8^+^ T cells recognize SARS-CoV-2 M, N, S proteins, nsp6, and ORF3a. ORF8 is able to downregulate MHC-I expression on diverse cell types. SARS-CoV-2 primarily induces S protein- and RBD-specific IgG, while IgM and IgA responses are lower

Apart from the S protein, other proteins, such as the N protein, M protein, non-structural proteins (nsps), and accessory proteins, may have the potential to serve as antigens. Indeed, viral proteins and their interactions with host factors were associated with imbalanced host immune responses, such as low type I interferons (IFN-I) and IFN-III levels, and elevated pro-inflammatory cytokine levels (Fig. 1a).^30,31^ Recent studies found that nsp13 of SARS-CoV-2 targeted the IFN pathway by associating with TBK1, and nsp15 interfered with this pathway by associating with RNF41. The open reading frame 6 (ORF6) protein interacted with the mRNA export factor NUP98-Rae1. ORF9b indirectly interacted with the mitochondrial antiviral signaling (MAVS) protein via its interaction with translocase of outer membrane 70 (Tom70).^32^ Moreover, ORF8 was shown to significantly downregulate the major histocompatibility complex class I (MHC-I) expression in diverse cell types via lysosomal degradation, thereby disrupting antigen presentation and impairing the cytotoxic T lymphocytes (CTLs)-mediated killing of virus-infected cells.^33^ Previous reports demonstrated that the CoV N protein induced protective specific CTLs.^34–37^ Moreover, NAbs titers significantly correlated with the number of N protein-specific T cells, suggesting that the production of NAbs might be linked with the activation of antiviral T cells.^28,38^ Another study reported that antisera to M proteins exhibited high neutralizing titers toward SARS-CoV infection, indicative of the importance of M protein for developing an effective protein-based vaccine.^39^ Recently, Grifoni et al. noticed that cluster of differentiation 4 (CD4)^+^ T-cell responses were primarily directed against the S, M, and N proteins and partially against nsp3, nsp4, and ORF8^40^ (Fig. 1b). Regarding CD8^+^ T-cell responses, the SARS-CoV-2 M and S proteins were strongly recognized, and significant reactivity was observed for other antigens, such as nsp6, ORF3a, and the N protein (Fig. 1b).^40^ The data suggests that beyond the S protein, the CD8^+^ T-cell response to SARS-CoV-2 elicited by an optimal vaccine may benefit from additional class I epitopes, such as those derived from the M, nsp6, ORF3a, and/or N protein. However, whether they can be used as the target antigen requires further investigation.

## The development of SARS-CoV-2 vaccines

### Inactivated vaccines and live-attenuated vaccines

**Fig. 2:**
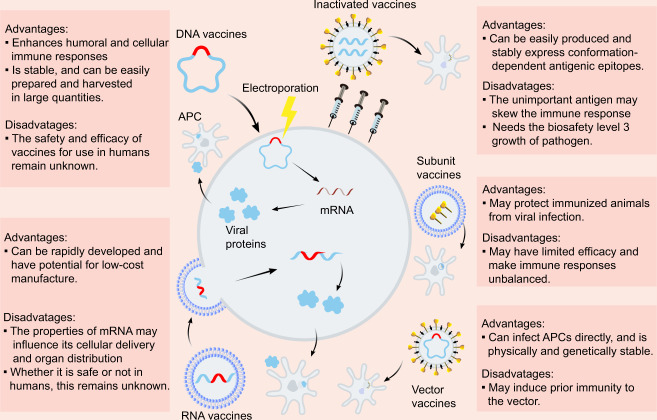
Overview of the diverse types of vaccines, and their potential advantages and disadvantages

Due to the urgent need to combat COVID-19, diverse SARS-CoV-2 vaccine types are currently under development, including inactivated vaccines, nucleic acid vaccines, adenovirus-based vector vaccines, and recombinant subunits vaccines (Fig. 2). Inactivated viruses are made non-infectious via physical or chemical approaches and are attractive because they present multiple viral proteins for immune recognition, have stable expression of conformation-dependent antigenic epitopes, and can be easily produced in large quantities.^41^ Purified inactivated viruses have been traditionally used for vaccine development and have been found to be effective in preventing viral diseases, such as influenza. The inactivated SARS-CoV-2 vaccine candidate, BBIBP-CorV, demonstrated potency and safety in animal models; thus, is expected to undergo further testing in clinical trials.^42^ Another study evaluating a purified inactivated SARS-CoV-2 virus vaccine candidate, PiCoVacc, showed the induction of NAbs against SARS-CoV-2 in mice, rats, and rhesus macaques with no notable cytokine changes or pathology observed in the macaques.^27^ The inactivated SARS-CoV-2 vaccine containing aluminum hydroxide developed by Sinovac has entered phase 3 clinical trials, with results from the phase 2 trial demonstrating that two doses of 6 μg/0.5 mL or 3 μg/0.5 mL of the vaccine were well-tolerated and immunogenic in healthy adults (Table 1).^43^ Phase 2 trial results of the inactivated SARS-CoV-2 vaccine, constructed by Wuhan Institute of Biological Products and Sinopharm, reported that the geometric mean titers (GMT) of NAbs were 121 and 247 at day 14 after 2 injections in participants receiving vaccine on days 0 and 14 and on days 0 and 21, respectively, displaying only transient and self-limiting adverse reactions.^44^

**Table 1:** The development of vaccine candidates in phase 3 clinical stage

Vaccine type	Vaccine	Developer	Clinical stage	Number of doses	Timing of doses	Reported results of clinical trials	Ref.
Inactivated vaccines	The inactivated SARS-CoV-2 vaccine with aluminum hydroxide	Sinovac	Phase 3	2	0, 14 days	Phase 2 trial showed that two doses of 6 μg/0.5 mL or 3 μg/0.5 mL of the vaccine were well-tolerated and immunogenic in healthy adults, with 3 μg dose eliciting 92.4% seroconversion under day 0, 14 schedule and 97.4% under day 0, 28 schedule.	^43^
	Inactivated	Wuhan Institute of Biological Products/Sinopharm	Phase 3	2	0, 14 or 0, 21 days	Phase 2 trial showed that the GMTs of NAbs were 121 and 247 at day 14 after 2 injections in participants receiving vaccine on days 0 and 14 and on days 0 and 21, respectively. Moreover, 7-day adverse reactions occurred in 6.0% and 19.0% of the participants receiving injections on days 0 and 14 vs on days 0 and 21.	^44^
	Inactivated	Beijing Institute of Biological Products/Sinopharm	Phase 3	2	0, 14 or 0, 21 days	N/A	N/A
RNA vaccines	BNT162b1	Pfizer/Fosun Pharma/BioNTech	Phase 3	2	0, 28 days	Phase 1/2 study showed that the vaccine caused mild to moderate local and systematic symptoms in most vaccinators and geometric mean neutralizing titers after the 10 and 30 µg dose 2 reached 1.8- to 2.8-fold that of COVID-19 convalescent sera panel.	^48^
	mRNA-1273	Moderna/NIAID	Phase 3	2	0, 28 days	Phase 1 study reported that the two-dose vaccine series was not seriously toxic and it could elicit NAbs and Th1-biased CD4^+^ T-cell responses.	^49^
Non-replicating vector vaccines	Adenovirus Type 5 Vector	CanSino Biological Inc./Beijing Institute of Biotechnology	Phase 3	1	N/A	Phase 2 trial showed that the vaccine at a dose of 5 × 10^10^ viral particles per mL was safer than the vaccine at 1 × 10¹¹ viral particles and elicited comparable immune response to it. However, high pre-existing Ad5 immunity reduced NAbs response and influenced T-cell immune response.	^55^
	ChAdOx1 nCoV-19	University of Oxford/AstraZeneca	Phase 3	1	N/A	Phase 1/2 trial reported that NAb responses were detected in 91% participants after a single dose when measured in MNA80 and in 100% participants when measured in PRNT50. After a booster dose, all participants had neutralizing activity. Local and systemic reactions, including pain, fever and muscle ache, could be reduced by paracetamol.	^59^
	Adeno-based (rAd26-S + rAd5-S)	Gamaleya Research Institute	Phase 3	2	0, 21 days	Phase 1/2 trial showed that administration of both rAd26-S and rAd5-S caused production of NAbs in 100% of participants on day 42 for both the lyophilized and frozen vaccine formulations. Cellular immune responses were detected in all participants at day 28. Moreover, the pre-existing immune response to the vectors rAd26 and rAd5 did not influence the titre of RBD-specific antibodies.	^57^
	Ad26COVS1	Janssen Pharmaceutical Companies	Phase 3	2	0, 56 days	Preclinical trials showed that a single immunization with an Ad26 vector encoding a prefusion stabilized S antigen triggered robust NAb responses and provided complete or near-complete protection in rhesus macaques. The immunogen contains the wildtype leader sequence, the full-length membrane-bound S, mutation of the furin cleavage site, and two proline stabilizing mutations.	^60^

Live-attenuated vaccines have demonstrated success in treating infections such as smallpox and poliomyelitis.^45^ Three SARS-CoV-2 live-attenuated vaccines that utilize a weakened virus as the antigen are under preclinical evaluation. However, such vaccines may revert to virulence in some cases. Although the virus itself can be used to develop vaccines, concerns have been raised that the inclusion of epitopes that do not induce NAbs or confer protection may skew the immune response, thereby requiring further investigation.

### Nucleic acid vaccines

Nucleic acid vaccines, such as mRNA vaccines and DNA vaccines, are other popular vaccine forms. These vaccines are delivered into human cells, where they will then be transcribed into viral proteins. Among the CoV proteins, S protein has been the most common candidate. mRNA vaccines represent a promising alternative compared to conventional vaccines due to their high potency, ability for rapid development, and cost-efficient production.^46,47^ However, the physiochemical properties of mRNA may influence its cellular delivery and organ distribution, and the safety and efficacy of mRNA vaccine use in humans remain unknown. Phase 1/2 studies investigating RNA vaccines (BNT162b1) targeting the RBD of the S protein, developed by Pfizer and BioNTech, reported that the vaccine caused mild to moderate local and systematic symptoms in most vaccinators, with the vaccine eliciting higher neutralizing titers after the second dose compared to the COVID-19 convalescent sera panel (Table 1).^48^ Phase 1 trial assessing mRNA-1273 that encoded the stabilized prefusion SARS-CoV-2 S protein demonstrated that the two-dose vaccine series did not cause severe adverse events and could elicit neutralization and Th1-biased CD4^+^ T-cell responses (Table 1).^49^ The lipid nanoparticles (LNP)-encapsulated mRNA vaccine encoding SARS-CoV-2 RBD called ARCoV conferred potent protection against SARS-CoV-2 in mice and non-human primates after two immunization doses. Moreover, it could be stored at room temperature, which would be more convenient for transportation and storage.^50^

DNA vaccines also have great therapeutic potential due to their ability to enhance T-cell induction and antibody production, the excellent biocompatibility of plasmid DNA, low-cost manufacturing, and their long shelf life.^51^ However, their disadvantage is that the DNA molecules must cross the nuclear membrane to be transcribed, and they generally have low immunogenicity. A study of various DNA vaccine candidates encoding different forms of the SARS-CoV-2 S protein discovered that vaccinated rhesus macaques were able to develop humoral and cellular immune responses and that vaccine-induced NAb titers were associated with protective efficacy.^52^ Notably, DNA vaccines induced type I helper T cells (Th1) instead of type II helper T cells (Th2) responses with no observed enhancement of clinical disease in rhesus macaques. However, a report concerning a MERS-CoV DNA vaccine observed NAbs in just half of all subjects and titers noticeably waned during the course of the study follow-up.^53^ Future studies should explore whether DNA vaccines are effective in inducing long-term NAbs and whether non-neutralizing antibody responses can confer protection or cause more severe disease.

### Vector vaccines

Vector vaccines are generally constructed from a carrier virus, such as an adeno or pox virus, and are engineered to carry a relevant gene from the virus, usually the S gene for CoVs. The key advantage of vector vaccines is that the immunogen is expressed in the context of a heterologous viral infection, which induces the innate immune responses required for the adaptive immune responses.^54^ Nevertheless, this strategy may induce prior immunity to the vector and are limited to presenting only a small number of CoV antigens to the host immune system. Clinical trials regarding an adenovirus type 5 (Ad5) vector vaccine carrying recombinant SARS-CoV-2, developed by CanSino Biological Inc. and Beijing Institute of Biotechnology, revealed that the vaccine at a dose of 5 × 10^10^ viral particles per mL was safer than the vaccine at 1 × 10^11^ viral particles, and elicited comparable immune response to it^55^ (Table 1). However, high pre-existing Ad5 immunity and increasing age reduced NAbs response and the pre-existing immunity might also influence T-cell immune response post-vaccination.^56^ Thus, further investigation is required to address these problems influencing vaccine efficacy. Phase 1/2 studies of a heterologous COVID-19 vaccine comprising a recombinant adenovirus type 26 (rAd26) vector and a recombinant adenovirus type 5 (rAd5) vector, both carrying the S gene of SARS-CoV-2, demonstrated that the pre-existing immune response to the vectors rAd26 and rAd5 did not influence the titre of RBD-specific antibodies (Table 1). Therefore, heterologous vaccination may be a good option to antagonize the negative impacts of immune response to vaccine vectors.^57^ Moreover, a phase 3 study was performed to determine the efficacy, safety, and immunogenicity of a chimpanzee adeno (ChAd)-vectored vaccine platform encoding a codon-optimized full-length SARS-CoV-2 S protein (ChAdOx1 nCoV-19). In a preclinical trial, SARS-CoV-2 genomic RNA was detected in nasal swabs from all rhesus macaques, with no discrepancy in viral load between nasal swabs on any day between ChAdOx1 nCoV-19-vaccinated and control animals, despite the lack of pneumonia and absence of immune-enhanced disease following viral challenge in vaccinated animals.^58^ However, in the phase 1/2 trial, ChAdOx1 nCoV-19 was shown to be safe, tolerated, and immunogenic. Moreover, local and systemic reactions, including pain, fever, and muscle ache, could be reduced by taking paracetamol^59^ (Table 1). Notably, safety is a crucial issue in vaccine development; therefore, greater emphasis on improving safety should be placed when testing the SARS-CoV-2 vaccines. Ad26COVS1 designed by Janssen Pharmaceutical Companies also entered the phase 3 clinical stage and its preclinical study showed that a single immunization with an Ad26 vector encoding a prefusion stabilized S protein triggered potent NAb responses and well protected the vaccinated rhesus macaques^60^ (Table 1).

### Subunit vaccines and virus-like particles vaccines

Subunit vaccines in which viral proteins are injected into the host have the potential to exhibit efficacy in protecting animals or human from viral infection. However, given that only a few viral components are included which do not display the full antigenic complexity of the virus, their protective efficacy may be limited and, in some cases, they may cause unbalanced immune responses.^61^ Yang et al. constructed a subunit vaccine composed of residues 319–545 of the SARS-CoV-2 RBD and produced it through the baculovirus expression system. The preclinical study reported that the vaccine could protect the non-human primates from SARS-CoV-2 infection with little toxicity^62^ (Table 2). Virus-like particles (VLPs) constitute another type of protein-based vaccines that are composed of proteins from the viral capsid.^63^ VLPs stimulate high immune responses due to their repetitive structures and are safer than several other vaccine platforms because they lack genetic material. The construction of VLPs similar to the authentic virus is a significant step in the development of an effective vaccine against infection. Several teams are currently working on engineering protein-based vaccines; however, the clinical results have not been published to date. Despite the fact that vaccine development is a lengthy and expensive process that typically involves multiple candidates and requires a lot of time to produce a licensed vaccine, it is vital to continue developing vaccines for the prevention and treatment of COVID-19.

**Table 2:** The development of vaccine candidates in phase 1 or phase 2 clinical stage

Vaccine type	Vaccine	Developer	Clinical stage	Number of doses	Timing of doses
The inactivated vaccines	Inactivated	Institute of Medical Biology, Chinese Academy of Medical Sciences	Phase 1/2	2	0, 28 days
	Inactivated	Research Institute for Biological Safety Problems, Rep of Kazakhstan	Phase 1/2	2	0, 21 days
	Whole-Virion Inactivated	Bharat Biotech	Phase 2	2	0, 14 days
	mRNA	Curevac	Phase 2	2	0, 28 days
RNA vaccines	mRNA	Arcturus/Duke-NUS	Phase 1/2	N/A	N/A
	LNP-nCoVsaRNA	Imperial College London	Phase 1	2	N/A
	mRNA	People’s Liberation Army Academy of Military Sciences/Walvax Biotech.	Phase 1	2	0, 14 or 0, 28 days
DNA vaccines	DNA plasmid vaccine with electroporation	Inovio Pharmaceuticals/ International Vaccine Institute	Phase 1/2	2	0, 28 days
	DNA plasmid vaccine + Adjuvant	Osaka University/ AnGes/ Takara Bio	Phase 1/2	2	0, 14 days
	DNA plasmid vaccine	Cadila Healthcare Limited	Phase 1/2	3	0, 28, 56 days
	DNA Vaccine (GX-19)	Genexine Consortium	Phase 1/2	2	0, 28 days
Non-replicating viral vector	Replication defective Simian Adenovirus (GRAd) encoding S	ReiThera/LEUKOCARE/Univercells	Phase 1	1	N/A
Replicating viral vector	Measles-vector based	Institute Pasteur/Themis/Univ. of Pittsburg CVR/Merck Sharp & Dohme	Phase 1	1 or 2	0, 28 days
	Intranasal flu-based-RBD	Beijing Wantai Biological Pharmacy/Xiamen University	Phase 1	1	N/A
Protein subunit	Full-length recombinant SARS-CoV-2 glycoprotein nanoparticle vaccine adjuvanted with Matrix M	Novavax	Phase 2	2	0, 21 days
	Adjuvanted recombinant protein (RBD-Dimer)	Anhui Zhifei Longcom Biopharmaceutical/Institute of Microbiology, Chinese Academy of Sciences	Phase 2	2 or 3	0, 28 or 0, 28, 56 days
	RBD-based	Kentucky Bioprocessing, Inc	Phase 1/2	2	0, 21 days
	S protein (baculovirus production)	Sanofi Pasteur/GSK	Phase 1/2	2	0, 21 days
	Recombinant trimeric subunit S protein vaccine	Clover Biopharmaceuticals Inc./GSK/Dynavax	Phase 1	2	0, 21 days
	Recombinant S protein with Advax™ adjuvant	Vaxine Pty Ltd/Medytox	Phase 1	1	N/A
	Molecular clamp stabilized S protein with MF59 adjuvant	University of Queensland/CSL/Seqirus	Phase 1	2	0, 28 days
	S-2P protein + CpG 1018	Medigen Vaccine Biologics Corporation/NIAID/Dynavax	Phase 1	2	0, 28 days
	RBD + Adjuvant	Instituto Finlay de Vacunas, Cuba	Phase 1	2	0, 28 days
	Peptide	FBRI SRC VB VECTOR, Rospotrebnadzor, Koltsovo	Phase 1	2	0, 21 days
	RBD (baculovirus production expressed in Sf9 cells)	West China Hospital, Sichuan University	Phase 1	2	0, 28 days
	SARS-CoV-2 HLA-DR peptides	University Hospital Tuebingen	Phase 1	1	N/A
VLP	Plant-derived VLP adjuvanted with GSK or Dynavax adjs.	Medicago Inc.	Phase 1	2	0, 21 days

## Neutralizing antibodies against SARS-CoV-2

**Fig. 3:**
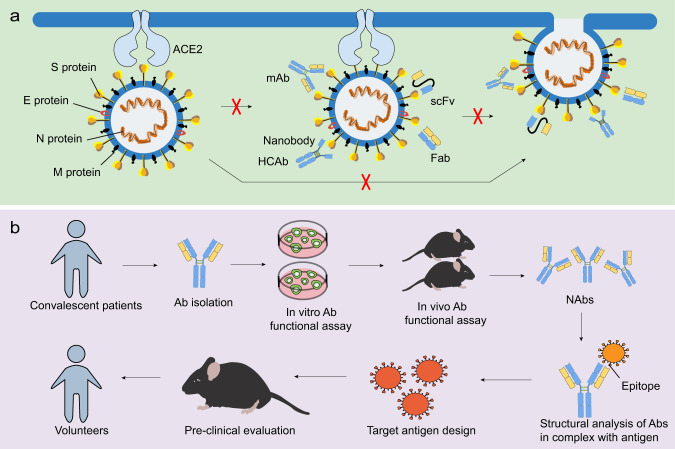
NAbs against CoVs and the scheme of Reverse Vaccinology 2.0. **a** NAbs, such as mAbs, single-domain antibodies, scFvs, and Fabs, are able to target viral proteins, with RBD being the most potent target. This process may further block receptor binding and membrane fusion, commonly via targeting the S1 and/or S2 subunit. **b** The scheme of Reverse Vaccinology 2.0. Antibodies are isolated from convalescent patients and tested for their efficacy in vitro and in vivo. NAbs are further studied in complex with the antigen. Identifying the epitopes may aid in immunogen design, which will later be evaluated in animal models and humans

**Fig. 4:**
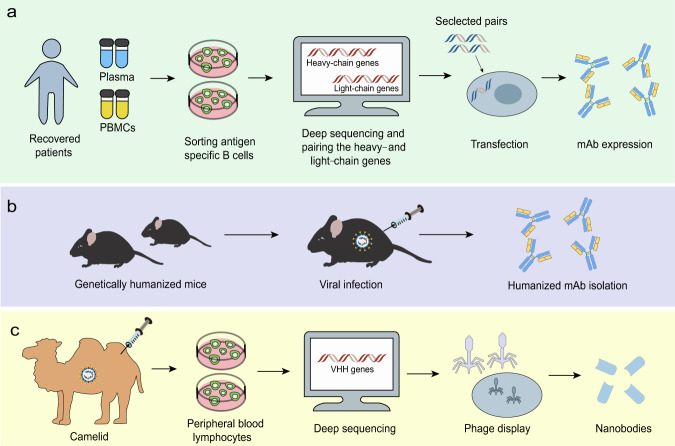
NAbs isolation strategies. **a** mAbs can be isolated from convalescent people previously infected with virus. After sorting antigen-specific B cells, deep sequencing can help pair the heavy- and light-chain genes. Selected pairs via functional screening can be used to produce mAbs. **b** Humanized mAbs can be isolated from immunized transgenic animal models, like mice. **c** Nanobodies can be constructed based on sequences of the camelid immunized with viral proteins and produced by phage carrying the VHH encoding sequences

NAbs play a critical role in controlling viral infection.^64^ The most commonly used antibody formats include monoclonal antibodies (mAbs), single-domain antibodies, single-chain variable fragments (scFvs), and functional antigen-binding fragments (Fabs) (Fig. 3a). Neutralizing monoclonal Abs can be isolated from recovered people previously infected with virus (Fig. 4a) or immunized transgenic animal models (Fig. 4b). NAbs, particularly those targeting the RBD of SARS-CoV-2, may serve as a promising therapeutic approach to viral infection^65,66^ (Table 3). Recently, three non-competing epitopes for the RBD (namely RBD-A, RBD-B, and RBD-C) have been identified, with RBD-A considered as the preferred target. RBD-A-directed NAb CC12.1 was shown to potently neutralize the pseudovirus.^67^ A cohort of NAbs were also shown to be able to bind the RBD and perturb the RBD-ACE2 interaction, such as BD-368–2, B38, H4, B5, CB6, and CV30.^68–74^ However, 47D11 and H2 did not compromise the spike-receptor interaction, although it was capable of binding to the epitope of the RBD of SARS-CoV-2.^69,75^ A study found that ACE2 competitor antibodies neutralized the viral infection by blocking ACE2 binding and inducing S1 dissociation, as well as demonstrating a weak association between antibodies potency and their binding affinity.^66^ However, a separate report revealed the correlation between serum RBD binding and virus neutralization.^67^ Additional efforts are required to characterize the factors that influence the neutralizing activities of NAbs. In light of the close relationship between SARS-CoV and SARS-CoV-2, scientists have attempted to identify SARS-CoV NAbs that cross-reacted with SARS-CoV-2. Antibodies derived from previously SARS-CoV-infected patients, such as S309, ADI-55689, and ADI-56046, were shown to cross-neutralize SARS-CoV-2.^66,76^ S309, which targeted a conserved glycan-containing epitope within the S protein, also displayed fragment crystallizable (Fc)-dependent effector mechanisms, such as antibody-dependent cell cytotoxicity (ADCC) and antibody-dependent cellular phagocytosis (ADCP).^76^ Moreover, a few NAbs targeted non-RBD regions (Table 3). For instance, CV1 and its clonal variant CV35 bound to an epitope distinct from the RBD and both exhibited lesser potency than CV30 that targeted the RBD region.^74^ Further efforts should focus on the identification of potent NAbs from recovered patients. Moreover, structural analysis using the Reverse Vaccinology 2.0 approach is expected to uncover the exact epitopes of NAbs in order to promote immunogen design and guide vaccine strategies (Fig. 3b).^77^

**Table 3:** Potential neutralizing antibodies targeting SARS-CoV-2

Ab type	Ab name	Ab source	Neutralizing mechanism	Ref.
mAb	CC12.1	Human	Targets the RBD-A epitope	^67^
	BD-368-2	Human	Overlaps with the ACE2 binding site	^68^
	B38, H4	Human	Show complete competition with ACE2 for binding to RBD	^69^
	B5	Human	Binds to the RBD but displays partial competition with ACE2	^69^
	H2	Human	Binds to the RBD but does not compete with ACE2 for RBD binding	^69^
	CB6	Human	Is overlapped with the binding epitopes of ACE2	^71^
	P2B-2F6	Human	Competes with ACE2 for binding to the RBD	^71^
	31B5	Human	Perturbs the ACE2-RBD interaction	^72^
	32D4	Human	Perturbs the ACE2-RBD interaction	^72^
	COVA1-18	Human	Perturbs the ACE2-RBD interaction	^73^
	COVA2-15	Human	Perturbs the ACE2-RBD interaction	^73^
	CV30	Human	Inhibits the S-ACE2 interaction	^74^
	CV1/CV35	Human	Binds to an epitope distinct from the RBD	^74^
	ADI-55689	Human	Binds at the edge of the ACE2 binding site	^76^
	ADI-56046	Human	Competes with both hACE2 and CR3022	^76^
	S309	Human	Targets a conserved glycan-containing epitope within S protein and shows Fc-dependent effector mechanisms	^66^
	47D11	Transgenic H2L2 mice	Binds to the conserved epitope of RBD without compromising spike-receptor interaction	^75^
	REGN10987 and REGN10933	Mice and human	Bind to two non-overlapping epitopes of the RBD	^89,90^
Fc-fusion	VHH-72-Fc	Camelid	Disrupts RBD dynamics and receptor binding	^81^
Nanobody	n3088, n3130	Human	Targets a cryptic epitope situated in RBD	^82^
scFv-Fc	5C2	Human	Inhibits ACE2 from binding to S protein	^84^

Apart from conventional antibodies, camelids generate heavy chain antibodies (HCAbs) composed of only two heavy chains with a single variable domain (VHH or nanobody) and two constant regions per chain. Nanobodies can be constructed based on sequences of the camelid immunized with viral proteins (Fig. 4c) or on human sequences. Compared to traditional antibodies, nanobodies have several unique characteristics due to their small size, including access to more epitopes, low production expense, and the possibility for large-scale production in prokaryotic expression systems.^78^ Moreover, nanobodies can be administered via an inhaler directly to the site of infection, which is particularly beneficial for the treatment of respiratory diseases.^79^ The disadvantages of utilizing nanobodies as therapeutics could be that they may show immunogenicity due to their camel derivation and lack Fc-mediated effector functions. However, humanization and the development of fully human antibodies could improve the nanobodies.^80^ Recently, the SARS-CoV RBD-directed single-domain antibody VHH-72 displayed cross-reactivity with the SARS-CoV-2 RBD and was capable of disrupting RBD-receptor-binding dynamics. Furthermore, a bivalent VHH-72-Fc construct exhibited neutralizing activity against SARS-CoV-2 S pseudoviruses.^81^ Analysis on fully human single-domain antibodies identified from an antibody library using SARS-CoV-2 S1 as panning antigens revealed that the antibodies n3088 and n3130 were able to neutralize SARS-CoV-2 by targeting a cryptic epitope situated in the spike trimeric interface, even though they are not able to compete with ACE2 for SARS-CoV-2 RBD binding.^82^ These two antibodies may serve as promising alternatives that may be less immunogenic than camelid or humanized nanobodies, given that they are entirely derived from human sequences.

In addition to the antibodies mentioned above, scFvs and Fabs hold promise for treating COVID-19, and have already demonstrated benefits in the context of fighting against SARS-CoV and MERS-CoV. The scFv 80R was shown to compete with ACE2 for interaction with the S1 subunit, and efficiently neutralized SARS-CoV in vitro.^83^ Recently, the RBD-specific scFv-human Fc 5C2 was found to effectively neutralize the SARS-CoV-2 S protein and inhibit ACE2 from binding to the S protein.^84^ Moreover, previous studies revealed that human mAbs or Fabs, such as MERS-27 and m336, could recognize epitopes on the RBD of MERS-CoV that overlapped with the dipeptidyl peptidase 4 (DPP4)-binding site and neutralized pseudotyped and/or live MERS-CoVs in vitro.^85,86^ The scFv and Fabs have short generation time, high antigen affinity, and structural stability.^87^ However, whether scFv and Fab are effective against SARS-CoV-2 requires further investigation.

Since there is a lack of effective therapies for treating a cohort of SARS-CoV-2-infected patients, further development of NAbs specifically targeted against SARS-CoV-2 may be worthwhile, as well as the continued investigation of NAbs against SARS-CoV and MERS-CoV that can cross-react with SARS-CoV-2. A SARS-CoV-2 variant carrying the Spike D614G mutation, which has greater infectivity, has become the dominant form in many geographic regions.^88^ It is noteworthy that CoVs have high mutation rates and NAbs have several limitations. As such, the use of NAbs that can synergistically recognize different epitopes warrants further research. The combination of REGN10987 and REGN10933 NAbs, which bound to two non-overlapping epitopes of the RBD, did not generate escape mutants.^89,90^ Antibody 553–15 identified by Wan et al. could substantially improve the neutralizing capacity of other NAbs they discovered.^91^ Nevertheless, the cocktail therapy approach is costly and may not induce long-term immune responses. Thus, continued efforts are required to improve the efficacy of cocktail therapy, and to assess whether it is practical and safe for clinical use.

## Safety concerns regarding vaccine development

**Fig. 5:**
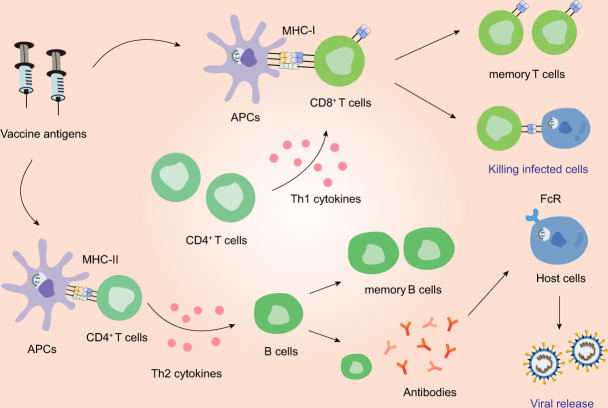
The immune responses induced by vaccines. Antigen-presenting cells (APCs) can process vaccine antigen and present it to CD8^+^ T cells and CD4^+^ T cells. CD8^+^ T cells can be stimulated by Th1 cytokines and in turn acquires the ability to attack the infected cells. Th2 cytokines can aid in the differentiation of B cells. The activated B cells can produce NAbs. However, imbalanced immune responses have the potential to cause pulmonary immunopathology, partially due to aberrant Th2 response or ADE

The most important criterion of vaccines is safety. Previous experience from the development of SARS-CoV and MERS-CoV vaccines has raised concerns of pulmonary immunopathology correlating with Th2 responses^65^ (Fig. 5b). Th2 is a subgroup of T cells that can secrete Th2-type cytokines, such as interleukin 4 (IL-4), IL-5, IL-10, and IL-13, and aberrant levels of Th2 cytokines can cause immune reactions that lead to eosinophil infiltrations. In murine models, four different SARS-CoV vaccines led to the occurrence of Th2-type immunopathology with high eosinophil infiltration, which served as a marker for Th2-type hypersensitivity.^92^ This was also observed in mice vaccinated with inactivated MERS-CoV vaccines which had eosinophil infiltrations, with the levels of IL-5 and IL-13 higher than those before vaccination.^93^ Moreover, it is proposed that the immunopathologic reaction following vaccination may be partially attributed to the presence of the N protein in the vaccine, but this requires further validation.^94,95^ Recent studies on cytokine changes in patients infected with SARS-CoV-2 also observed increased secretion of Th2 cytokines, which might contribute to the lung immunopathology.^96–98^ Thus, controlling the T-cell response must be considered when designing vaccines against SARS-CoV-2.

While the humoral immune response induced by vaccines may represent a potent approach of conferring protection against CoV infection, an abnormal antibody response may also result in physical deterioration of patients (Fig. 5b). In SARS-CoV-infected macaque models, vaccine-induced S-specific IgG resulted in severe acute lung injury (ALI) because IgG disturbed the inflammation-resolving response of macrophages and the blockade of Fc gamma receptor (FcγR) reduced such influence.^99^ Moreover, deceased patients displayed higher titers of NAbs and faster NAb responses which dropped more quickly than in recovered patients during the acute infection, potentially triggering a systematic breakdown of the immune system and exerting the immunopathologic effects on the lung and spleen.^99,100^ Consistently, patients severely infected with SARS-CoV-2 frequently exhibited more robust IgG responses and increased antibodies titers, which linked with the worst clinical condition and suggested antibody-dependent enhancement (ADE) of SARS-CoV-2 infection.^101,102^ Whether SARS-CoV-2 vaccines will cause abnormal antibody responses is currently unknown and additional research is required to address the potential lung damage caused by SARS-CoV-2 vaccine candidates.

Age is known to influence vaccine immunity. Vaccinated aged animals that were challenging to immunize also displayed eosinophilic immune pathology in the lungs. Worse still, neutralizing titers were significantly reduced in aged vaccinated groups compared to young groups.^95,103^ In essence, elderly populations with underlying diseases including diabetes, hypertension, and cardiovascular disease are at high risk for infection by SARS-CoV-2.^52,104^ Given the severity of disease in elderly people, aged animal models are essential for the preclinical validation of vaccines.

## Potential strategies to optimize vaccines

### Antigen design

The identification of immunodominant B- and T-cell epitopes that trigger protective immune responses in the host is critical for effective vaccine design. Given that SARS-CoV-2 strains shared ~79% identity with SARS-CoV at the whole-genome level, several recent studies predicted a series of B-cell and T-cell epitopes from the SARS-CoV-2, based on the experimentally-determined SARS-CoV epitopes.^13,105^ Ahmed et al. identified a set of T-cell epitopes, 49 liner B epitopes, and 6 discontinuous B epitopes that were identical to SARS-CoV-2 proteins, and the majority of the epitopes were derived from the S- or N protein.^106^ Comparison of the epitopes identified by homology to the SARS-CoV-derived epitopes with the epitopes identified by epitope predictions, identified 12 SARS-CoV-2 T-cell epitopes, three linear B-cell epitopes, and two conformational B epitope regions as promising targets for SARS-CoV-2 immune recognition.^107^ Via an extensive immunoinformatics-based approach, Mukherjee et al. identified 25 immunodominant epitopes from SARS-CoV-2 proteins: 4 epitopes in the M protein, 8 epitopes in the N protein, and 13 epitopes in the S protein. Among these, the seven epitopes: M protein 165–181 and 306–322, N protein 314–330, S protein 817–833, 891–907, 897–913, and 1182–1209, that covered over 87% of the world’s population were found to be non-allergen, non-toxic, and with a low risk of causing autoimmune reactions.^108^ Thus, they may serve as candidates for designing SARS-CoV-2 vaccines. Another eight immunodominant CD4^+^ T-cell epitopes have been suggested for use in a subunit vaccine, to potentially elicit effective T- and B-cell responses. They are distributed across the S protein (232–246 and 233–247), E protein (55–69, 56–70, and 57–71), and M protein (97–111, 98–112, and 99–113).^109^ These predictions warrant further investigation and may aid effective vaccine design against SARS-CoV-2.

Optimally designed vaccines aim to maximize immunogenicity to protein domains that play a critical role in protective immunity while excluding unnecessary protein domains that may cause autoimmunity or even enhanced infectivity. Experiments conducted on rhesus macaques demonstrated that the SARS-CoV S protein peptides 471–503, 604–625, and 1164–1191 induced antibodies that conferred protection, while peptide 597–603 induced antibodies that enhanced infection through an epitope sequence-dependent (ESD) mechanism.^110^ Thus, it may be important to eliminate epitopes that enhance viral infection in order to prepare a safe vaccine. The postfusion conformation may expose the non-neutralizing epitopes and distract the host immunity.^111^ Therefore, minimizing the number of the postfusion S2 trimers may improve the efficacy of vaccines, which warrants further investigation. Recently, Yarmarkovich et al. identified 65 peptides dissimilar to self-peptides that were predicted to target the vulnerabilities of SARS-CoV-2 and stimulate adaptive immunity and proposed their use in DNA or mRNA vaccines.^112^ It was also noticed that most SARS-CoV-2 compositions were immunogenically silent on MHC-I and MHC-II, and should thus be excluded from vaccine development.^112^ Taken together, it is essential to identify epitopes capable of inducing potent immune responses while decreasing the likelihood of inducing autoimmunity.

**Fig. 6:**
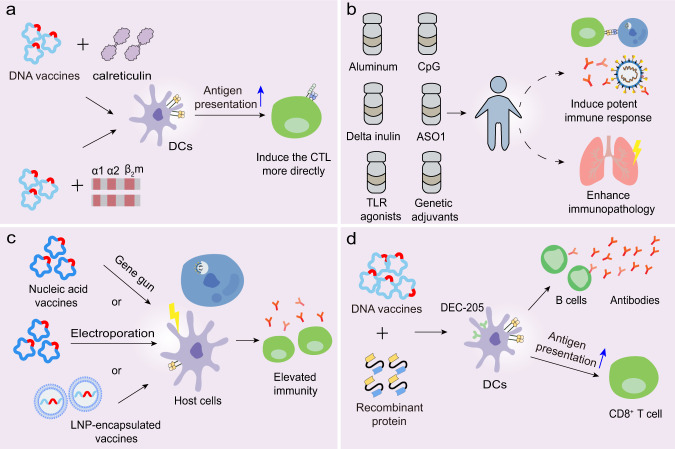
Potential strategies to optimize vaccines. **a** DNA vaccines linked with calreticulum or the cDNA of human β_2_-microglobulin and the α-1 and α-2 domains of MHC-I heavy chain can facilitate antigen presentation and induce the CTL response more directly. **b** Adjuvants have the potential to promote the immune response against CoVs, although several are involved in the immunopathology. **c** Certain types of vaccines can be delivered into host cells via gene gun, electropolaration, or LNP, thereby resulting in a broader protective immunity. **d** DNA vaccines linked with recombinant protein targeting the DC molecules, DEC-205, can induce potent humoral and cellular immune responses

Furthermore, structural antigen design plays a significant role in vaccine efficacy. The S protein variant named HexaPro contains four beneficial proline substitutions (F817P, A892P, A899P, A942P) and two proline substitutions in the S2 subunit, thereby increasing protein yields and stability.^113^ The high yield of stabilized prefusion S proteins may promote industrial production of subunit vaccines and nucleic acid vaccines. The RBD engineered as a tandem repeat single-chain dimmer (sc-dimer) is proposed for the development of vaccines against betacoronaviruses, which may improve the immunogenicity for antibody responses and neutralization. Two immunizations of RBD-sc-dimers were shown to maximize NAb titers for vaccines against MERS-CoV, SARS-CoV-2, and SARS-CoV.^114^ Moreover, DNA vaccination with antigen linked to calreticulin (CRT) dramatically enhanced MHC-I presentation of the linked antigen to CD8^+^ T cells and generated potent humoral and cellular immune responses in vaccinated C57BL/6 mice (Fig. 6a).^37^ Cheung et al. developed an approach in which a DNA vaccine expressed an antigenic peptide from the SARS-CoV N protein that linked with the cDNA of human β_2_-microglobulin and the α-1 and α-2 domains of the human MHC-I heavy chain (Fig. 6a).^115^ This method reduced the uncertainty of antigen processing in the antigen-presenting cells (APCs) and induced the CTLs more directly. The optimal structural design of immunogens deserves further investigation to enhance the antigen presentation capacity and induction of efficient immune responses.

### Adjuvants

Another way of improving SARS-CoV-2 vaccines is by adding adjuvants to the vaccine formulations. Adjuvants are able to enhance the immunogenicity of the co-injected vaccine antigens, polarize the immune response toward the desirable response and increase the human immune response. Adjuvants, such as aluminum, MF59, and the adjuvant system (AS) series adjuvants developed by GlaxoSmithKline (GSK), are typically utilized in the development of various vaccines (Fig. 6b).^92,116^ In murine models, alum-formulated vaccines were associated with significantly increased lung eosinophilic immunopathology, while delta inulin adjuvant enhanced T-cell IFN-γ responses rather than inducing the eosinophilic infiltration, despite increasing the frequency of IL-4-secreting T cells.^117^ This data suggested that the inadequate Th1 response might contribute to the lung immunopathology and that alum might not be suitable for use in CoV vaccines. However, macaques, administered with novel BBIBP-CorV that was mixed with aluminum hydroxide, exhibited normal lungs with focal mild histopathological changes in a few lobes. Indeed, aluminum has long been utilized as an adjuvant and has demonstrated efficacy and safety in diverse vaccines.^118^ Whether alum is appropriate for use in SARS-CoV-2 vaccines needs additional research. The combination of two adjuvants, alum and CpG, was reported to induce a balanced or Th1-biased immune response in mice.^119,120^ A separate study showed that CpG was able to halt long-term anti-S protein T and B-cell memory responses, but promoted IgG2, IgG3, and IFN-γ production in the short term; however, this requires further validation.^117^ In addition, no lung immunopathology was observed among hamsters vaccinated with a SARS-CoV whole virus vaccine with GSK adjuvant ASO1 which was able to induce Th1-type immune responses.^121^ Given that vaccines may induce lung injury due to Th2-type responses, and some adjuvants promote a Th2-type biased immune response, a Th1-type adjuvant is proposed to alleviate the potential immunopathology, and is worth further investigation.^103,122^ Moreover, MF95, an oil-in-water emulsion adjuvant, was found to augment the immunogenicity of MERS-CoV RBD-based subunit vaccines, thereby inducing robust IgG and NAb responses and protecting mice against viral infection. Hence, whether MF95 is an optimal adjuvant for the SARS-CoV-2 subunit vaccines deserves studying.^123^

Toll-like receptor (TLR) agonists can stimulate innate immune responses and elicit adaptive immune responses, thereby improving vaccine efficacy. TLR agonists were shown to inhibit the skewing of immune responses toward the Th2 response and reduce excess eosinophilic infiltration in the lungs.^124,125^ Moreover, genetic adjuvants encoding transcriptional factors functioned to stimulate APCs and enhance the immune responses, which could be co-expressed in nucleic acid vaccines. The immunogenicity of DNA vaccines could be elevated by co-transfection of IFN regulatory factors (IRFs), such as IRF-3 and IRF-7.^126,127^ Moreover, co-injection of the plasmid encoding the virus-induced signaling adapter (VISA) and a DNA vaccine encoding influenza protein, co-activated IRF and NF-κB transcription factors and augmented IFN-γ-specific T-cell responses.^128^ Whether this methodological approach is suitable for SARS-CoV-2 vaccines deserves further investigation. Collectively, it is important to select appropriate adjuvants when developing optimal vaccines against SARS-CoV-2, and additional trials are needed to evaluate the efficacy of adjuvants and their potential to induce immunopathology in humans.

### Several promising delivery approaches

To ensure that vaccines trigger protective responses, it is critical to adopt effective approaches to deliver antigen into the host cells. The gene gun serves as a practical method to deliver RNA and DNA.^129,130^ A previous study demonstrated that the delivery of DNA vaccines to dendritic cells (DCs) via gene gun, primed CD8^+^ CTL responses against viral infection.^130^ Moreover, electroporation increased the cellular uptake of DNA or self-amplifying RNA, thereby causing elevated immune responses (Fig. 6c).^131,132^ DCs are professional APCs of the immune system, and vaccines targeting DCs may promote antigen representation and facilitate the immune responses. Immunization with DCs coated with SARS-CoV peptide from the SARS-CoV S protein induced virus-specific CD8^+^ T cells in BALB/c mice, resulting in earlier virus clearance and increased survival (Fig. 6c).^133^ The DC-targeting protein that specifically bound to the DC surface molecule, DEC-205, could potentially be used for the delivery of DNA vaccines directly to DCs. This would provide the capacity to improve the immunogenicity and antiviral activity of DNA vaccines, as seen with the hepatitis B virus (HBV) DNA vaccine (Fig. 6d).^134,135^

Apart from the aforementioned techniques, potential effective delivery may also be achieved by administering the combination of nucleic acids with compounds such as lipids and polymers. In recent years, LNPs have become an attractive delivery approach in vaccine development (Fig. 6c). The LNPs are generally composed of four lipid components, namely an ionizable cationic amino lipid, phospholipids, cholesterol, and lipid-linked polyethylene glycol (PEG). The ionizable amino lipid significantly aids the intracellular delivery of encapsulated nucleic acid and promotes its endosomal release after LNP endocytosis. The phospholipids play a role in forming a lipid bilayer, cholesterol functions to stabilize the LNP and PEG increases the shelf life. Antigens, such as nucleic acid and viral subunit, encapsulated within an LNP displayed improved immunogenicity, and resulted in protective immunity.^136,137^ Moreover, LNP on its own elicited a biased Th2-type immune response, whereas LNP plus TLR9 agonist immune-modulatory oligonucleotides (IMO) induced a more dominant Th1-type B-cell response.^137^ Thus, LNP in combination with certain adjuvants may also have potential to boost T-cell and B-cell responses against SARS-CoV-2, which warrants further exploration.

## Conclusions and perspectives

The widespread threat of SARS-CoV-2 to humans has spawned challenges to develop safe and effective antiviral drugs and vaccines for preventive use. Currently, several clinical trials have shown that ritonavir, lopinavir, chloroquine, and hydroxychloroquine had little benefit for COVID-19 treatment. A randomized, controlled and open-label trial revealed that ritonavir and lopinavir did not clearly shorten the time to clinical improvement compared to the standard care.^138^ Both chloroquine and hydroxychloroquine had the potential to affect the corrected QT (QTc) interval, and chloroquine is not recommended for severe patients.^139–141^

Several antibodies have been identified to target different domains of SARS-CoV-2 and are effective in neutralizing SARS-CoV-2. These antibodies may have the potential to treat SARS-CoV-2-infected patients, and future work to define these antibody epitopes will further aid vaccine development. The experimental and clinical results of some vaccine candidates, such as BBIBP-CorV and PiCoVacc, were reported, with most vaccines showing neutralizing capacity. For vaccine development, it is critical to generate protective T- and B-cell immune responses. The S protein has been shown to be the most potent antigen for SARS-CoV and MERS-CoV vaccines, and we hypothesize this may be similar for SARS-CoV-2 vaccines. However, the immunopathology induced by SARS-CoV or MERS-CoV vaccines was observed in animal models, which might be attributed to ADE, an aberrant Th2 response partially due to the N protein, as well as other unknown reasons. The mechanisms underlying this immunopathology deserve further investigation, which may provide instructive guidance for the future development of SARS-CoV-2 vaccines. Apart from immunopathology, other important questions remain to be addressed, such as how to protect the population vulnerable to lethal human CoVs, such as the elderly, and how best to provide protection against variant and heterologous CoV strains. Recently, human ACE2 transgenic mice were developed that could be infected by SARS-CoV-2 and generated typical pathology that were similar to those of COVID-19 patients.^142,143^ Rhesus macaques infected by SARS-CoV-2 also exhibited humoral and cellular immune responses and were protected from re-challenge.^144^ In essence, it is equally important to identify the ideal animal model for evaluating potential SARS-CoV-2 vaccines.

Herein, we reviewed current vaccine strategies of several pathogenic viruses with the aim to improve vaccine efficacy and safety against SARS-CoV-2. Antigen design plays a significant role in maximizing the immunogenicity. It is necessary to include the important epitopes while excluding the unimportant ones. Moreover, the structure design of the immunogen requires additional research. Employing a suitable delivery system is also critical for vaccine efficacy. Determining which method works best depends on many factors, including the types of vaccines and vaccination routes. Furthermore, adjuvants should be added to the various types of vaccines to enhance immunogenicity; therefore, the selection of appropriate adjuvants is crucial for developing SARS-CoV-2 vaccines. Until now, only several studies had reported the immune responses induced by SARS-CoV-2 vaccine candidates. Further trials must test the safety and efficacy of vaccines and search for effective approaches to optimize the vaccines. In conclusion, we hope the insights provided above will aid in the development of SARS-CoV-2 vaccines.
